# Estrogen-like and tissue-selective effects of 7-methoxycoumarin from *Ficus umbellata* (Moraceae): an in vitro and in vivo study

**DOI:** 10.1186/s12906-017-1895-9

**Published:** 2017-08-02

**Authors:** Stéphane Zingue, Thomas Michel, Chantal Beatrice Magne Nde, Amstrong Nang Njuh, Julia Cisilotto, Derek Tantoh Ndinteh, Colin Clyne, Xavier Fernandez, Tânia Beatriz Creczynski-Pasa, Dieudonné Njamen

**Affiliations:** 1 Department of Life and Earth Sciences, Higher Teachers’ Training College, University of Maroua, P.O. Box 55, Maroua, Cameroon; 2 Department of Pharmaceutical Sciences, Health Sciences Centre, Federal University of Santa Catarina, CEP, Florianópolis, Santa Catarina 88040-900 Brazil; 3 Department of Applied Chemistry, Faculty of Science, University of Johannesburg, Doornfontein, 2028 South Africa; 4 Institute of Chemistry of Nice, Faculty of Science, University Côte d’Azur, UMR CNRS 7272, Valrose Park, Cedex 2 Nice, France; 5 Hudson Institute of Medical Research, Clayton, VIC 3168 Australia; 6 Department of Animal Biology and Physiology, Faculty of Science, University of Yaoundé 1, P.O. Box 812, Yaounde, Cameroon

**Keywords:** *Ficus umbellata*, 7-methoxycoumarin, Phytoestrogens, E-screen assay, Uterotrophic assay

## Abstract

**Background:**

*Ficus umbellata* is a medicinal plant previously shown to endow estrogenic properties. Its major component was isolated and characterized as 7-methoxycoumarin (MC). Noteworthy, coumarins and the respective active metabolite 7-hydroxycoumarin analogs have shown aromatase inhibitory activity, which is of particular interest in the treatment of estrogen-dependent cancers. The present work aimed at evaluating the estrogenic/antiestrogenic effects of MC in vitro and in vivo.

**Methods:**

To do so, in vitro assays using E-screen and reporter gene were done. In vivo*,* a 3-day uterotrophic assay followed by a postmenopausal-like rat model to characterize MC as well as *F. umbellata* aqueous extract in ovariectomized Wistar rats was performed. The investigations focused on histological (vaginal and uterine epithelial height) and morphological (uterine wet weight, vagina stratification and cornification) endpoints, bone mass, biochemical parameters and lipid profile.

**Results:**

MC induced a significant (*p* < 0.05) MCF-7 cell proliferation at a concentration of 0.1 μM, but did not inhibit the effect induced by estradiol in both E-screen and reporter gene assays. In vivo, MC treatment did not show an uterotrophic effect in both rat models used. However, MC (1 mg/kg) induced a significant increase (*p* < 0.01) of vaginal epithelial height. No significant change was observed with MC in abdominal fat weight, serum lipid levels and bone weight.

**Conclusion:**

These results suggest that MC has a weak estrogenic activity in vitro and in vivo that accounts only in part to the estrogenicity of the whole plant extract. MC could be beneficial with regard to vagina dryness as it showed a tissue specific effect without exposing the uterus to a potential tumorigenic growth.

**Electronic supplementary material:**

The online version of this article (doi:10.1186/s12906-017-1895-9) contains supplementary material, which is available to authorized users.

## Background

Menopause is an inevitable phenomenon occurring naturally in women, characterized by a drop in estrogen levels and a definite cessation of menstruation [1]. It sets around the age of 50 years or following total ovariectomy and affects all women, independent of race and/or social strata [2]. Menopause marks the end of the reproductive life span of women and is often associated with a variety of physiological disorders like hot flushes, depressions and body weight increase. Although such disorders are not life threatening; the social life, as well as, the productivity of the women affected might be negatively impacted [3]. In the long term, estrogen deficiency affects bone density [4] and the cardiovascular system [5]. Hormone replacement therapy (HRT) has therefore been proposed as a solution to postmenopausal symptoms. It is especially effective for the treatment of severe vasomotor and estrogen-deficiency mucosal symptoms, as well as in slowing down the rate of bone demineralization [6]. However, Women’s Health Initiative and the British Million Women Studies reported that HRT is associated with higher incidence of mammary cancers and arteriosclerotic complications such as heart attack and stroke [7]. This raises questions about the exclusive beneficial effects of HRT [8]. Emerging evidence tends to support the short-term use of HRT for severe menopausal symptoms and its avoidance for long-term prophylaxis [9]. Many menopausal women have therefore turned to non-hormonal therapies [10]. Nowadays, there is the vigorous promotion of plant-derived phytoestrogens in a bid to seek for alternatives to HRT. The structural similarity of phytoestrogens to endogenous estradiol permits them to bind and activate estrogen receptors in mammals. The considerable scientific effort is being placed in a search for phytoestrogens that could exhibit optimal estrogenic activity, i.e. with positive effects on bone health while having little effect on uterine or breast tissues [11]. *Ficus umbellata* Vahl (Moraceae) is a tree that grows in tropical areas; commonly called “Tol’l” in “Ewondo” or “Mewed” in “Guiziga”. The bark of *F. umbellata* is used to treat menopause related physiological disorders [12]. In our previous work, aqueous and methanol extracts of *F. umbellata* transactivated the Estrogen Receptor α (ERα) in a reporter gene assay and induced significant estrogen-like effects on estrogen target organs (uterus, vagina and mammary gland) in rats. Furthermore, it significantly decreased the frequency of hot flushes in experimental rats. Its major component was isolated and characterized as 7-methoxycoumarin (MC) [13]. Coumarins consist of a group of compounds characterized by 1,2-benzopyrone or benzopyran-2-ones, which are extensively studied. A number of coumarins exhibit interesting pharmacological activities and are therefore of therapeutic use [14]. Indeed, some coumarins and their active metabolite 7-hydroxycoumarin analogs have shown aromatase inhibitory activity, which is of particular interest in the treatment of estrogen-dependent cancers (ovaries, uterus and breast cancers) [15, 16]. Preliminary in vitro tests performed with this compound showed that it failed to transactivate ERα and ERβ in a reporter gene assay [13]. In the present study, in depth in vitro estrogenic assay and a 3-day uterotrophic assay in ovariectomized adult rats were performed to characterize the effects of MC. Thereafter, the effects of MC as well as *F. umbellata* aqueous extract were evaluated using a postmenopausal-like rat model.

## Methods

### Chemicals

Mass Spectroscopy (MS) grade methanol, acetonitrile (ACN), water and formic acid (FA) were purchased from Sigma-Aldrich (Saint-Quentin Fallavier, France). Fetal bovine serum (FBS) and antibiotics were purchased from GIBCO (Grand Island, NY). The 17β-estradiol benzoate [(Estr-1,3,5(10)-trien-3,16α,17β-triol); purity ≥98%] was obtained from Sigma-Aldrich (Hamburg, Germany). Estradiol valerate (Progynova® 2 mg) was purchased from DELPHARM (Lille, France). The 2-[4-(2-hydroxyethyl)piperazin-1-yl]ethane sulfonic acid (HEPES, purity ≥99.5%) was purchased from Ludwig Biotecnologia Ltda (Alvorada, RS, Brazil). Trypan blue, Sulforodamine B, Serum Replacement 2 and cell culture mediums were purchased from Sigma-Aldrich (St. Louis, MO, USA). Genistein was obtained from “Extrasynthese®” (Genay, France).

### Plant material and preparation of *F. umbellata* extracts

Stem barks of *F. umbellata* were harvested in Yaounde (Centre region, Cameroon) in September 2013. This botanical sample was authenticated by Mr. Victor Nana, a botanist at the National Herbarium of Cameroon (HNC) by comparison to the specimens deposited under the voucher number 99/HNC. After drying under the shade in an aerated place for 2 weeks, the well-dried stem barks of *F. umbellata* were pulverized by electronic grinding. Then, 2 kg of powder was macerated in water at room temperature (5 L of solvent **×** 3, 48 h per extraction). Thereafter solutions were filtered through Whatman paper N°4 and evaporated using an oven with ventilation (40 °C, during 48 h) to yield 229.8 g of aqueous crude extract. Likewise, 2.7 kg of the powder was macerated in 95% methanol at room temperature (5 L of solvent × 3, 48 h per extraction). The combined solutions were evaporated under reduced pressure (337 mbar at 40 °C) using a rotary evaporator to yield 162 g of a methanol crude extract (MeOH).

### Isolation of 7-methoxycoumarin (MC)

The different Ultra Performance Liquid Chromatography (UPLC) chromatograms of *F. umbellata* aqueous and methanol extracts showed that MC is their major compound. The MC was then isolated after a bio-guided fractionation of *F. umbellata* methanol extract as previously reported [13]. The physicochemical properties of MC are summarized in Table 1.

**Table 1:** General information on 7-methoxycoumarin isolated from *F. umbellata*

Code and names	Crystal color	Structure, molecular weight and formula
7-methoxycoumarin Methylumbelliferone 7-methoxy-2H-chromen-2-one MC	White	Molecular weight = 176.1 M Molecular formula = C_10_H_8_O_3_

### UPLC-ESI-MS analyses of *F. umbellata*

Analyses of *F. umbellata* extracts were performed by UPLC-high resolution electrospray ionization MS (HRESIMS) on an ACQUITY UPLC®/Xevo™ G2 QTof (Waters, USA). All separations were performed on an Acquity UPLC BEH C-18 column (100 mm × 2.1 mm I.D., 1.7 μm) at 25 °C with a flow rate of 0.400 mL/min. A guard column (5 mm × 2.1 mm, 1.7 μm) with the same stationary phase was placed before the column. The mobile phase consisted of water +0.1% FA (solvent A) and ACN + 0.1% FA (solvent B) and was used in multistep gradient mode. The gradient was operated as follows: 0–1 min, 5% B; 1–9 min, 5–40% B; 9–15 min, 40–100% B. The injection loop was set at 0.5 μL and 10 °C was used as the sample manager temperature. The standard Acquity PDA module was used for online UV detection in the range of 200–700 nm range, with a resolution of 1.2 nm and a sampling rate of 10 spectra/s. The HRMS and HRMS/MS data were acquired in negative mode with a mass range of 100–1500 m/z. The ESI conditions operated were as follows: source temperature 120 °C, desolvation temperature 500 °C; capillary voltage 1.5 KV, cone voltage 10 V. Nitrogen was used as a cone (10 L/Hr) and desolvation gases (1000 L/Hr). Lockspray flow rate was set at 20 μL/min and lockspray capillary at 2.5 KV. For the HRMS/MS acquisitions, a method including the detection (full scan) and fragmentation of the most intense peaks per scan was used. The collision energy varied from 10 to 35 V.

### In vitro experiments

#### Cell line systems

The MCF-7 – human ER-positive breast adenocarcinoma cell line was obtained from the Rio de Janeiro Cell Bank (Federal University of Rio de Janeiro, Brazil). Human Embryonic Kidney 293 T (HEK 293 T) cell line expressing the SV40 large T-antigen was obtained from ATCC (The Global Bioresource Center, Australia). The luciferase reporter assay, ERα and ERβ expression plasmids were kindly provided by Dr. Simon Chu (Hudson Institute of Medical Research, Australia). Cells were transfected using Lipofectamine Reagent obtained from Invitrogen (Sydney, Australia).

#### Cell culture

MCF-7 cells were cultured in RPMI-1640 medium supplemented with fetal bovine serum (FBS) 10%. Cell cultures were also supplemented with penicillin 100 U/mL, streptomycin 100 μg/mL and HEPES 10 mM. They were maintained at 37 °C in a CO_2_ 5% humidified atmosphere and pH 7.4. Every 2 days, cells were passaged by removing 90% of the supernatant and replaced by fresh medium. Viable cells (a minimum of 95%) were checked at the beginning of the experiment by Trypan Blue dye exclusion test.

HEK293T cells were routinely maintained in Dulbecco’s Modified Eagle Medium (DMEM) supplemented with sodium pyruvate 1 mM, glutamine 4 mM, 4.5 g/l of glucose, and fetal bovine serum 10% in a humidified atmosphere at 37 °C in a CO_2_ 5%. Prior to transfection, the media was replaced by the 5% Charcoal Stripped Serum DMEM phenol free. Cells were then seeded at the density of 50,000 per well in a 24 well-plate format. The next day, they were transfected with appropriate plasmids in the starving media O/N and treated in the same media.

#### E-screen assay

The E-screen MCF-7 cells proliferation assay was performed as described by Resende et al. [17]. Briefly, cells were trypsinized and seeded in 24-well plates at an initial concentration of 5 × 10^4^ cells per well in RPMI supplemented with FBS 10%. After 24 h of incubation (37 °C, CO_2_ 5%) to permit their adhesion, cells were washed with phosphate-buffered saline-PBS (NaCl 137 mM; KCl 2.7 mM; Na_2_HPO_4_ 10 mM_;_ KH_2_PO_4_ 1.8 mM_;_ pH 7.4) and the Serum Replacement 2 (0.5×) supplemented phenol red-free RPMI was substituted for the seeding medium. A stock solution of 7-methoxycoumarin 100 mM prepared in DMSO was then added to the experimental medium in order to reach concentrations from 1 × 10^−8^ to 1 × 10^−5^ M. The DMSO concentration of 0.01% was fixed based on the final volume on different wells. For antiestrogenic evaluation, before cell incubation, 17β-estradiol 1 × 10^−8^ M was added to the wells. Cells treated with DMSO (0.01%) and FBS 10% in RPMI served as solvent and medium controls, respectively. The assay was stopped after 144 h by removing the medium from the wells, fixing the cells with cold trichloracetic acid 10% and incubated at 4 °C for 1 h. Thereafter, cells were washed four times with tap water and dried. Cells were stained for 30 min with sulforhodamine-B (SRB) 0.057% (*w*/*v*) which was dissolved in 1% acetic acid, rinsed four times with acetic acid 1% and air-dried. Bound dye was solubilized with Tris base 10 mM (pH 10.5) in a shaker. Finally, aliquots were read in a Biotek EL800 absorbance reader (Winooski, USA) at 510 nm. The results related to estrogenic activity were expressed as mean ± standard error of the mean (SEM) of the proliferative effect (PE), which was calculated according to Schiliro’et al. [18]: *PE = max cell number of sample/cell number of DMSO control*. The estrogenic activity of a sample was determined as the relative proliferative effect (RPE%). The RPE compares the maximum proliferation induced by a sample with that induced by 17β-estradiol: *RPE% = [PE for sample/PE for 17β-estradiol]* × *100* [17].

#### Transfections and luciferase assays

As far as the MC failed to transactivate estrogen receptors α and β, in the Human Embryonic Kidney 293 T cell line (HEK293T) transiently transfected with adequate plasmids using Lipofectamine Reagent as thoroughly described by Zingue et al. [13]. The present experiment was aimed to evaluate the possible antiestrogenic effects of this compound in the same cell system and at the concentrations used in our previous study [13]. Briefly, HEK293T cells were transiently transfected with 200 ng of estrogen α-receptor plasmid or estrogen β-receptor expression plasmid, together with the double estrogen response element (ERE) and a luciferase reporter [250 ng (ERE)2-tk-Luc] plasmid kindly provided by Dr. Simon Chu (Hudson Institute of Medical Research, Melbourne, Australia) and β-galactosidase reporter plasmid using Lipofectamine Reagent (Invitrogen, Mulgrave, Australia). They were then treated with different concentrations (1 × 10^−8^ to 1 × 10^−5^ M) of the MC in combination with E2 for 24 h prepared from a 100 mM stock dissolved in 70% Ethanol. E2 alone or in combination with ICI 182,780 were used as positive control. Reporter gene assays in HEK293T-ERα cells and HEK293T-ERβ cells were done using a commercial kit (Promega, Melbourne, Australia) according to the manufacurer’s instructions. Luciferase activity was measured and normalized toward β-galactosidase activity determined by using the 2-nitrophenyl b-d-galactopyranoside (ONPG) method (Sigma-Aldrich, Sydney, Australia).

### In vivo experiments

#### Animals

Healthy juvenile female Wistar rats aged 3 months (~150 g) were obtained from the breeding facility of the Laboratory of Animal Physiology, University of Yaounde I (Cameroon). Animals were housed in clean plastic cages at room temperature (around 25 °C) under natural illumination (approx. 12 h light/dark). The animals had free access to a standard soy-free rat chow (Table 2) and water.

**Table 2:** Rat chow composition

Content	Mass (g/100 g)	Percentage
Corn	36.467	36.5%
Bone flour	14.587	14.6%
Wheat	36.467	36.5%
Fish flour	4.862	4.9%
Crushed palm kernel	7.293	7.3%
Sodium chloride (NaCl)	0.303	0.3%
Vitamin complex (Olivitazol®)	0.018	0.02%

#### Ethical consideration

The housing of animals and all experiments were approved by the Cameroon Institutional National Ethic Committee, which adopted all procedures recommended by the European Union on the protection of animals used for scientific purposes (CEE Council 86/609; Reg. no. FWA-IRD 0001954).

#### Determination of doses

For the in vivo experiments, estradiol benzoate (E2B) and faslodex were administered subcutaneously at the doses of 2 μg/kg and 300 μg/kg, respectively as described by Zingue et al. [19]. The well-known phytoestrogen genistein was administered at a dose of 10 mg/kg [20]. Based on the amount of the 7-methoxycoumarin isolated from *F. umbellata* methanol extract, the quantity contained in the optimal dose (200 mg/kg) of this extract was found to be around 1 mg/kg. This dose was framed between the lowest dose of 0.1 mg/kg and the highest dose of 10 mg/kg.

#### The 3-day uterotrophic assay

**Fig. 1:**
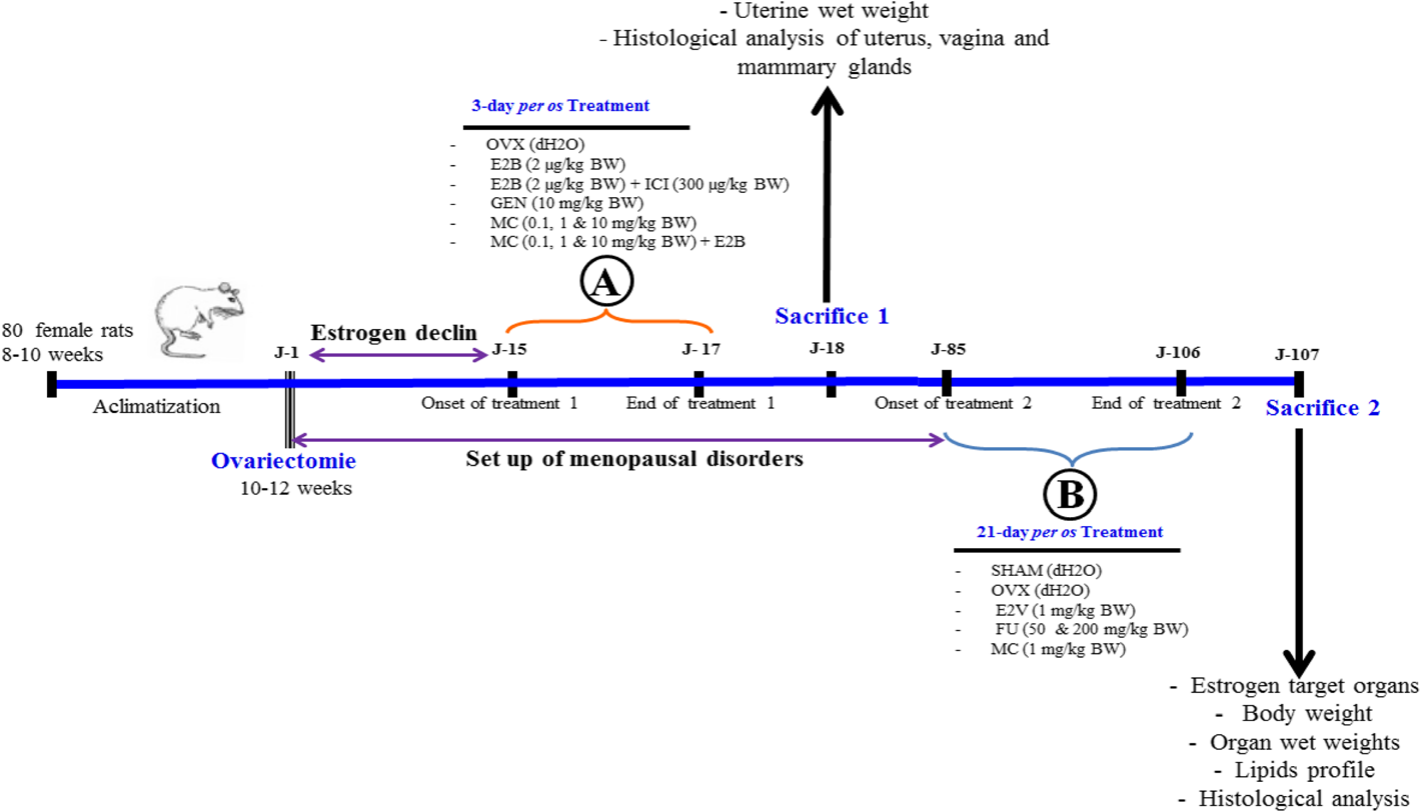
Scheme of the experimental protocol. Experimental protocol for a 3-day uterotrophic assay (**a**) and 21-day treatment in a postmenopausal-like rat model (**b**)

In this part of the work (Fig. 1a), 50 female Wistar rats aged 2.5 months were bilaterally ovariectomized (OVX) using the dorsal approach under Diazepam and ketamine anesthesia (respectively 10 mg/kg and 50 mg/kg BW; i.p.). After 14 days of endogenous hormonal decline, animals were randomly allocated into 10 groups of five animals each (*n* = 5). Group I received vehicle (corn oil) and served as negative control. Group II received estradiol Benzoate (E2B) as standard drug at the dose of 2 μg/kg per day. Group III received estradiol Benzoate (E2B, 2 μg/kg) co-administered with the pure antiestrogen faslodex (ICI 182,780; 300 μg/kg). Group IV received genistein as phytoestrogen control at the dose of 10 mg/kg. Group V, VI and VII received 7-methoxycoumarin (MC) at doses of 0.1, 1 and 10 mg/kg, respectively. The remaining three groups (group VIII, IX and X) were co-treated with estradiol Benzoate (E2B, 2 μg/kg) and MC at doses of 0.1, 1 and 10 mg/kg, respectively. Estradiol benzoate, faslodex (ICI, 182,780), MC and genistein were dissolved in vehicle (corn oil). All treatments were given by subcutaneous route (0.3 mL/150 g) for 3 consecutive days. Twenty-four hours after the last injection, animals were euthanized. The uterine wet weight, uterine and vaginal epithelial heights were assessed as described before by Zingue et al. [13].

#### Postmenopausal-like rat model

Five animals were sham-operated under Diazepam and ketamine anesthesia (respectively 10 mg/kg and 50 mg/kg BW; i.p.) and 25 other rats were bilaterally ovariectomized as described above (Fig. 1b). Eighty four (84) days after ovariectomy after the onset of postmenopausal-like conditions in rat [21], animals were randomly distributed into six groups of 5 animals each (*n* = 5). Group I (SHAM) was sham operated rats and received distilled water. Group II to VI were ovariectomized rats treated as follows: Group II received distilled water as a negative control (OVX). Group III served as positive control and received estradiol valerate (1 mg/kg). Group IV received the MC at the dose of 1 mg/kg and remaining Group V and VI the *F. umbellata* aqueous extract at the doses of 50 and 200 mg/kg. All substances were dissolved in distilled water and administered by oral gavage route (2 mL/250 g) for 21 consecutive days. Throughout the experiment, animals were weighed once every week in order to determine the weight gain. Twenty-four hours after the last treatment, animals were euthanized and blood samples were collected and centrifuged at 3000×g for 15 min and serums were stored at −4 °C for further analysis. The uterine, vagina, mammary gland, abdominal fat, liver, lungs, kidneys, adrenergic gland, femur and tibia were collected, cleaned of the superficial fatty layer and weighed. Femurs were dried at 110 °C for 12 h and weighed. All collected organs were fixed in 10% formalin for histological analysis.

### Histological analysis

The histomorphology of the uterus, vagina, femur, liver, lungs and kidneys was performed from 5-μm sections of paraffin-embedded tissues following hematoxylin-eosin staining. Organs were photographed at 40  × magnification using the complete Zeiss equipment consisting of a microscope Axioskop 40 connected to a computer where the image was transferred, and analyzed with the MRGrab1.0 and Axio Vision 3.1, both Zeiss (Hallbermoos, Germany) software’s’.

### Biochemical analysis

Total-cholesterol, HDL-cholesterol, and triglycerides blood levels were determined enzymatically using reagent-kits purchased from Biolabo (France). The artherogenic index was calculated as total cholesterol on HDL cholesterol.

### Statistical analysis

Results were presented as means ± standard error of the mean (SEM). In vitro experiments were performed in triplicates and repeated three times. Data analysis was performed with GraphPad Prism 6.0 software, using the ANOVA test followed by the Dunnett’s post hoc test to compare each treatment with a control group. Differences were considered significant at a probability level of 5% (*p* < 0.05).

## Results

### Phytochemical analysis of *F. umbellata* extract by UHPLC-HRMS/MS

**Fig. 2:**
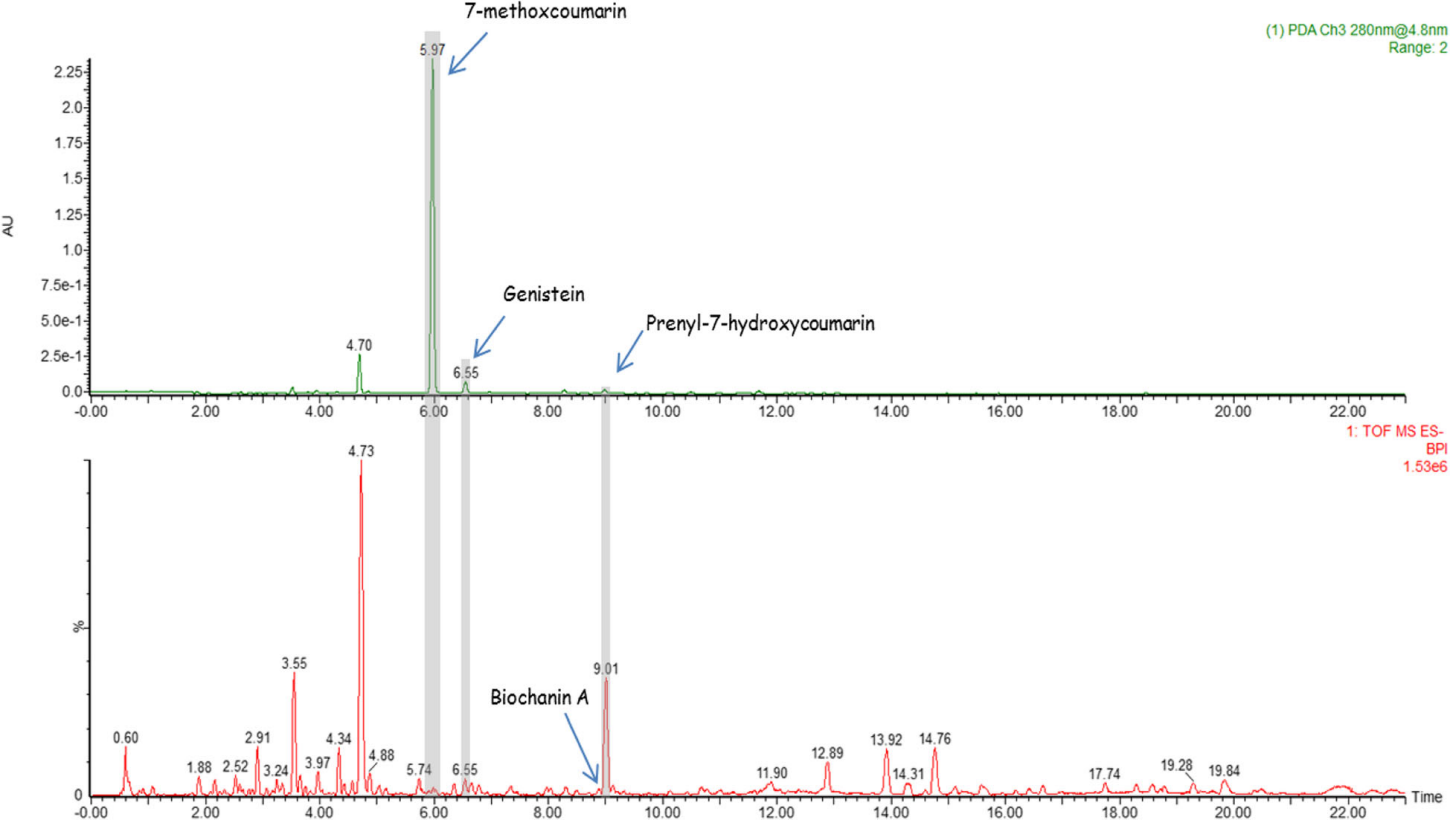
Analysis of *Ficus umbellata* aqueous extract. UHPLC–ESI-HRMS base peak chromatogram of *Ficus umbellata* aqueous extract in the negative ionisation mode

A qualitative analysis of the *F. umbellata* extract was performed using UHPLC-HRMS/MS by electrospray ionization in negative mode. Compound identification was based on their retention time, elemental composition (EC), monoisotopic mass of the pseudomolecular ion (m/z), RDBeq values, and their major HRMS/MS fragments. All well resolved peaks in TIC was selected and possible EC were calculated. The compounds detected in the methanol extract are summarized in Additional file 1: Table S1 and only eventual estrogenic compounds have been depicted in Fig. 2. A total of 40 secondary metabolites were detected in the active extract of *F. umbellata* belonging to various chemical classes including hydroxybenzoic acids, hydroxycinnamic acids, coumarins, flavonoids and triterpene derivatives. Among them, 4 were identified or putatively characterized based on the dereplication strategy as eventual estrogenic compounds (Fig. 2). In this study, we confirmed the presence of 7-methoxycoumarin at *m/z* 175.0384 (Rt 6.0 min) already described in our previous work as the major component of *F. umbellata* [13], but also other coumarins such as dihydroxycoumarin at m/z 177.0184 (Rt 6.35 min) and prenyl-7-hydroxycoumarin at 229.0865 (Rt 9.01 min). Genistein (Rt 6.55 min, m/z 269.0446) and biochanin A (Rt 8.89 min, *m/z* 283.0607) already described in *F. carica* [22] were identified based on their MS/MS fragmentation patterns.

### In vitro estrogenicity assessment

#### E-screen assay

**Fig. 3:**
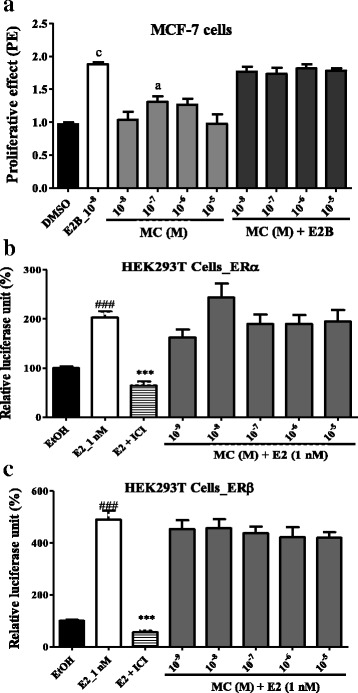
Effects of 7-methoxycoumarin (MC) on cell proliferation and on estrogen receptors. MCF-7 cells proliferation (**a**); activation of estrogen α (**b**) and β (**c**) receptors in HEK293T cells. The relative MCF-7 cells yields (PE) were measured in the presence of DMSO (0.01%), 17β-estradiol (10 nM) and MC. The effect of 7-methoxycoumarin on estrogen α and β receptors activity in the transiently transfected HEK293T-ERα and HEK293T-ERβ cells was investigated by measuring reporter gene-coupled luciferase activity. The relative luciferase units (RLU) were measured in the presence of EtOH (0.1%), E2 (10 nM), 7-methoxycoumarin (MC) co-treated with E2 (**a** & **b**). PE = max cell number of sample/cell number of DMSO control. *** *p* < 0.001 as compared to EtOH control; ### *p* < 0.001 as compared with E2 control. ^a^p < 0.05, ^c^p < 0.001 as compared to DMSO control

In the Fig. 3a is shown a significant (*p* < 0.001) increase by 17-β estradiol-induced in MCF-7 cells proliferation. The MC induced a significant (*p* < 0.05) MCF-7 cells proliferation only at the concentration of 0.1 μM as compared to DMSO control. No antiestrogenic activity was noted with all tested concentrations of MC in this assay.

#### Transactivation assay

The Fig. 3b and c showed that MC failed to inhibit the transactivation of ERα as well as ERβ induced by estradiol (E2). No significant change was observed with the co-treatment MC plus estradiol, suggesting that it has no antiestrogenic properties.

### In vivo estrogenicity assessment

#### Effects on uterus

**Fig. 4:**
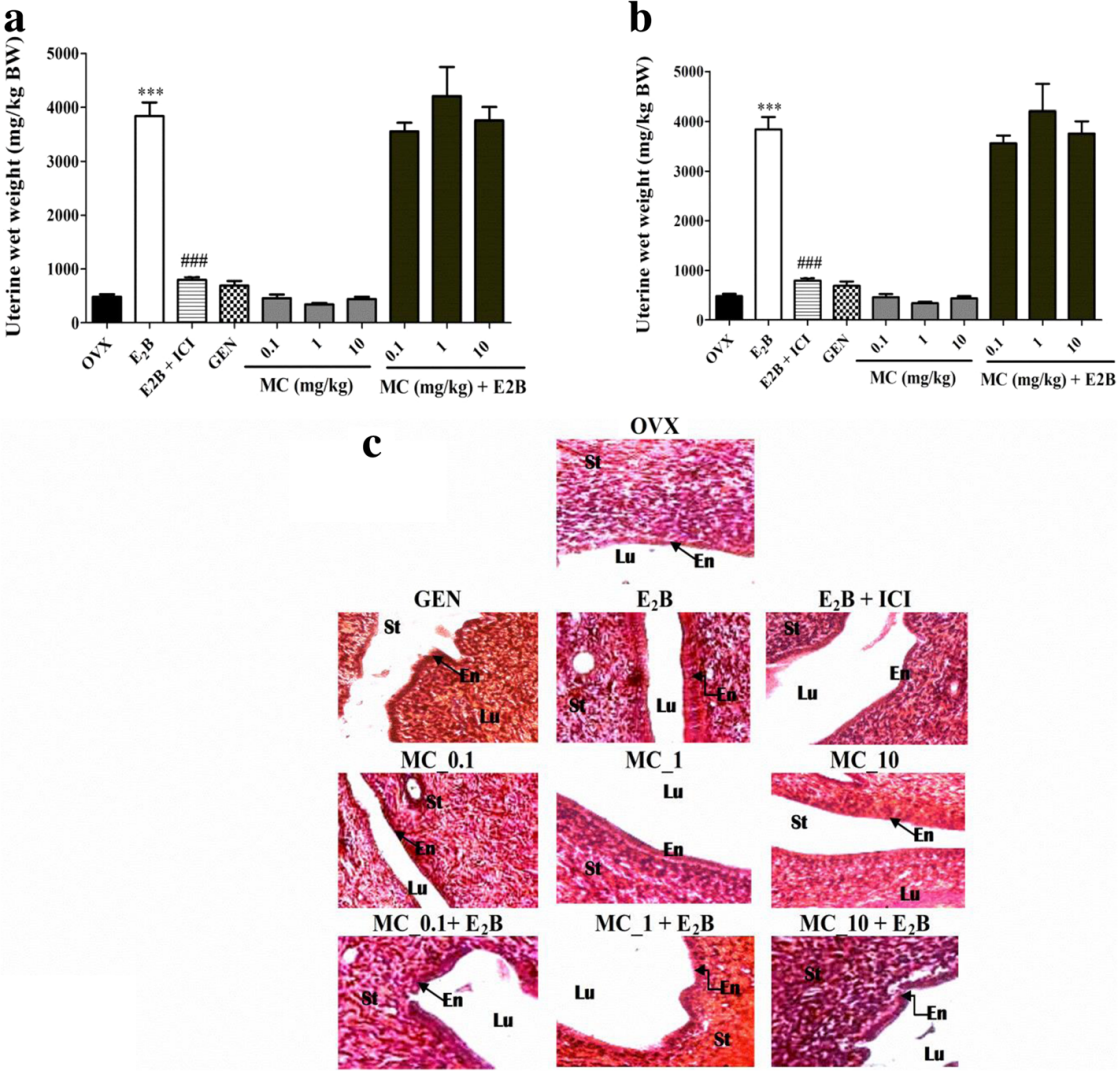
Analysis of the rat uterus. Graphic representations of uterine wet weight (**a**), uterine epithelial height (**b**) and microphotographs of H&E-stained sections (400×) of uterus (**c**). OVX = Ovariectomized rat treated with vehicle; E_2_B = Ovariectomized rat treated with estradiol benzoate at the dose of 2 μg/kg. E_2_B + ICI = Ovariectomized rat co-administered with estradiol benzoate (1.75 μg/kg) and ICI, 182.780 (300 μg/kg). GEN = Ovariectomized rat treated with genistein at the dose of 10 mg/kg BW. MC = Ovariectomized rat treated with 7-methoxycoumarin at the doses of 0.1, 1 and 10 mg/kg BW. MC + E_2_B = Ovariectomized rat co-administered with estradiol benzoate (1.75 μg/kg) and MC at the doses of 0.1, 1 and 10 mg/kg BW. *** *p* < 0.001 as compared to OVX group. ## *p* < 0.01, ### *p* < 0.001 as compared to E_2_B group. Lu = lumen of uterus; En = endometrium; St = Stroma

Figure 4 depicts that E2B induced a significant (*p* < 0.001) increase in the uterine wet weight as well as in the uterine epithelial height as compared to the OVX group. The pure antiestrogen faslodex significantly (*p* < 0.001) abolished the effects of E2B. Genistein, a well characterized phytoestrogen did not exert significant changes in uterine wet weight whereas, it induced a significant (*p* < 0.05) increase in uterine epithelial height. No estrogenic effects were noted following the treatment with MC in the uterus at all tested doses. However, MC (0.1 mg/kg) induced a significant (*p* < 0.05) decrease in the endometrium height when co-administered with E2B (Fig 4b).

#### Effects on vagina

**Fig. 5:**
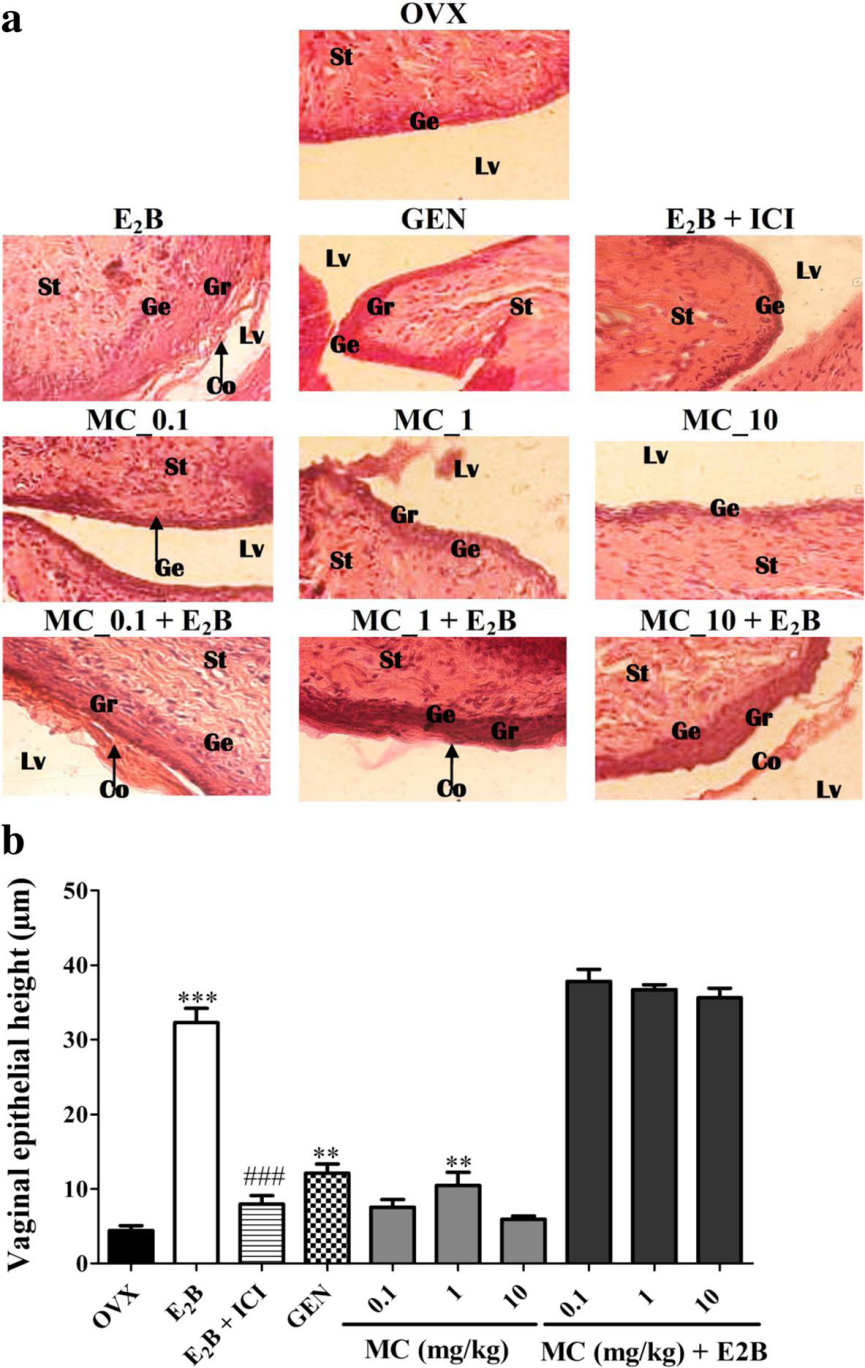
Analysis of the rat vaginas. Microphotographs of H&E-stained sections (400×) of vagina (**a**) and graphic representation of the vaginal epithelial height (**b**). OVX = Ovariectomized rat treated with vehicle; E_2_B = Ovariectomized rat treated with estradiol benzoate at the dose of 2 μg/kg. E_2_B + ICI = Ovariectomized rat co-administered with estradiol benzoate (1.75 μg/kg) and ICI, 182.780 (300 μg/kg). GEN = Ovariectomized rat treated with genistein at the dose of 10 mg/kg BW. MC = Ovariectomized rat treated with 7-methoxycoumarin at the doses of 0.1, 1 and 10 mg/kg BW. MC + E_2_B = Ovariectomized rat co-administered with estradiol benzoate (1.75 μg/kg) and 7-methoxycoumarin at the doses of 0.1, 1 and 10 mg/kg BW. ** *p* < 0.01; *** *p* < 0.001 as compared to OVX group. ### *p* < 0.001 as compared with E_2_B group. Lu = lumen of uterus; En = endometrium; St = Stroma

The effects observed following a 3-day treatment with different substances on the vagina are shown in Fig. 5. There was a significant increase in the vaginal epithelium height with E2B (*p* < 0.001) and genistein (*p* < 0.01) treatments, while co-treatment of the E2B and Faslodex significantly (*p* < 0.001) abolished the effects of estradiol on vaginal epithelium. The MC induced a significant increase (*p* < 0.01) in the vaginal epithelium height only at the dose of 1 mg/kg. No antiestrogenic activity was noted on vagina epithelium with MC.

### Effect of 7-methoxycoumarin and *F. umbellata* extract on postmenopausal symptoms

#### Effects on estrogen target organs

Effects of different treatments on estrogen target organs in postmenopausal-like conditions are summarized in Table 3. An 84-day post-ovariectomy induced a significant (*p* < 0.001) reduction in uterine wet weight, uterine and vaginal epithelial heights. E2V significantly (*p* < 0.001) increased the uterine wet weight as well as uterine and vaginal epithelial heights. There was no change on the uterine wet weight as well as the uterine epithelial height upon administration of *F. umbellata* aqueous extract. Interestingly, *F. umbellata* aqueous extract at both doses (50 and 200 mg/kg) as well as MC (1 mg/kg) significantly (*p* < 0.01) increased vaginal epithelial height as compared to control OVX (Table 3).

**Table 3:** Effects of 7-methoxycoumarin (MC) and *F. umbellata* aqueous extract on estrogen target organs and lipids profile on postmenopausal-rat model after 21 days treatment

		Ovariectomized rats				
Items	SHAM	OVX	E2V	FU 50	FU 200	MC
Estrogen target organs						
Uterine wet weight (mg/kg)	1921.36±185.26	356.9±19.05^###^	1288.42±136.9***	546.12±44.24	349.69±7.69	409.83±15.81
Uterine epithelial height (μm)	6.71±0.36	3.06±0.16^###^	5.27±0.15***	3.6±0.37	3.84±0.18	3.39±0.20
Vaginal epithelial height (μm)	19.06±0.11	2.54±0.56^###^	14.25±0.55***	6.24±0.20**	7.86±0.01**	8.56±1.39***
Lipids profile						
Total cholesterol level (mg/dL)	67.92±6.27	93.9±2.29^##^	56.98±11.27*	83.01±10.27	56.53±4.99*	79.24±11.67
Triglycerides levels (mg/dL)	47.96±3.75	62.96±7.35^#^	44.66±2.02*	63.01±6.46	49.48±1.67	56.08±4.26
HDL-Cholesterol level (mg/dL)	46.62±0.91	44.91±0.34^#^	45.94±0.54*	45.1±0.38	45.60±0.66*	45.33±0.44
Atherogenic risk	1.28±0.20	1.63±0.15^#^	1.19±0.15*	1.84±0.22	1.19±0.21 *	1.69±0.22

SHAM = Sham operated rats treated with vehicle as normal control; OVX = Ovariectomized rats treated with vehicle as negative control; E_2_V = Ovariectomized rats treated with estradiol valerate at the dose of 1 mg/kg BW; FU 50 and 200 = Ovariectomized rats treated with *F. umbellata* aqueous extract at the doses of 50 and 200 mg/kg BW, respectively; MC = Ovariectomized rats treated with 7-methoxycoumarin at the dose of 1 mg/kg BW. ^#^ *p* <0.05, ^##^ *p* < 0.01, ^###^ *p* <0.001 as compared to SHAM group. *p <0.05, **p < 0.01, ***p <0.001 as compared to OVX group

#### Fasting serum lipids

A slight but significant (*p* < 0.05) increase in fasting serum total cholesterol levels and triglycerides levels (Table 3) was observed in animals of OVX group compared to sham operated animals, while the HDL-cholesterol levels significantly (*p* < 0.01) decreased (Table 3). Hence, the atherogenic risk, calculated as the ratio of total cholesterol to HDL-cholesterol increased. The E2V group (1 mg/kg BW) significantly (*p* < 0.05) decreased triglycerides and total cholesterol levels, while it increased HDL-cholesterol level after 21 days of treatment. *F. umbellata* aqueous extract at the dose of 200 mg/kg BW on the other hand decreased total cholesterol and increased HDL-cholesterol levels as compared to the control. Moreover, the artherogenic risk was significantly decreased by E2V and the methanol extract of *F. umbellata* at the dose of 200 mg/kg BW. The 7-methoxycoumarin did not induce significant changes in fasting serum lipids (Table 3).

#### Effects on body weight and relative organ weights

Body weight of all animals increased after the ovariectomy. Table 4 displays only the variation of body weight during the period of treatment. A significant (*p* < 0.05) increase in the body weight between the onsets of treatment until the end with OVX group was noted. A slight decrease in body weight was noted with estradiol treatment (E2V). *F. umbellata* aqueous extract prevented increase in the body weight.

**Table 4:** Body weight and relative organ weights after 21 days of treatment with 7-methoxycoumarin (MC) and *F. umbellata* aqueous extract on postmenopausal-rat model

Organs (mg/kg)	Ovariectomized rats					
	SHAM	OVX	E2V	FU 50	FU 200	MC
Body weight (g)						
Initial	166.11 ± 8.74	196.21 ± 5.96	223.4 ± 11.16	221.4 ± 9.99	227.2 ± 6.37	217.4 ± 9.63
Final	170.12 ± 9.07	228.25 ± 9.87^**#**^	227.8 ± 10.38	213.1 ± 12.30	232.2 ± 4.88	231.2 ± 10.30
Abdominal fat	214,781.26 ± 7565.50	24,764.24 ± 3112.63	126,762.1 ± 12,637.27**	171,691.87 ± 7204.37	204,538.59 ± 10,865.97*	159,774.48 ± 6658.63
Liver	270,365.48 ± 8253.71	264,972.88 ± 3943.36	283,443.82 ± 7685.54	262,571.47 ± 9392.70	238,002.87 ± 6425.51	235,675.89 ± 5742.40
Lungs	118,498.50 ± 15,472.69	94,800.44 ± 6201.97	72,416.58 ± 7256.60	95,271.092 ± 15,943.46	83,943.78 ± 1247.63	89,067.78 ± 6817.63
Kidneys	64,222.43 ± 4210.62	52,320.42 ± 2500.44	55,721.72 ± 2277.40	59,531.12 ± 3155.58	56,560.25 ± 1821.70	56,512.05 ± 2116.07
Femur	38,986.17 ± 3280.98	34,515.25 ± 916.01	37,866.95 ± 2081.32	45,917.25 ± 6570.63	52,231.77 ± 2007.19**	33,434.41 ± 2449.05
Dried Femur	18,786.86 ± 22.23	16,838.91 ± 1007.69	17,670.96 ± 341.22	21,179.00 ± 1341.27 *	24,415.28 ± 852.49 **	16,766.41 ± 902.75
Tibia	22,684.56 ± 1637.99	17,455.86 ± 1167.04	19,311.75 ± 831.75	21,544.21 ± 917.37	30,168.61 ± 1688.17 **	21,109.35 ± 2933.98
Adrenergic	3467.83 ± 199.62	2561.80 ± 289.87	2337.06 ± 237.95	2834.97 ± 192.60	2693.05 ± 116.49	3066.73 ± 211.96

SHAM = Sham operated rats treated with vehicle as normal control; OVX = Ovariectomized rats treated with vehicle as negative control; E_2_V = Ovariectomized rats treated with estradiol valerate at the dose of 1 mg/kg BW; FU 50 and 200 = Ovariectomized rats treated with *F. umbellata* aqueous extract at the doses of 50 and 200 mg/kg BW, respectively; MC = Ovariectomized rats treated with 7-methoxycoumarin at the dose of 1 mg/kg BW. ^##^ *p* < 0.01 as compared to SHAM group. *p < 0.05 **p < 0.01 as compared to OVX group; #*p* < 0.05 Body weight at the end of treatment compared to itself at the beginning of the treatment

No change was noted in relative weights of liver, lungs, kidneys and adrenergic glands (Table 4). However, a significant increase in femur wet (*p* < 0.01) and dried (*p* < 0.001) weights as well as in tibia wet weight (*p* < 0.01) with *F. umbellata* treatment at both doses was noted. In addition, a significant decrease of abdominal fat was observed in rats treated with E2V (*p* < 0.01) and *F. umbellata* aqueous extract at the dose of 200 mg/kg (*p* < 0.05).

#### Effects on the microarchitecture of some organs

No alterations in the microarchitecture of liver, lungs and kidneys were noted in this work (Additional file 1: Figure S2). However, the femur microarchitecture of OVX rats showed bone marrow disparities into the trabecular network. E2V and *F. umbellata* aqueous extract (200 mg/kg) treatments prevented bone resorption, evidenced by the inhibition of bone marrow loss into the trabecular network. MC did not induce significant change in the bone microarchitecture.

## Discussion

Coumarins comprise of compounds characterized by 1,2-benzopyrone or benzopyran-2-ones, which are extensively studied. Many valuable biological activities have been assigned to coumarins [14]. The 7-methoxycoumarin (MC) used in this study was isolated as the major component of *F. umbellata*. This compound failed to transactivate estrogen receptors (ERα and ERβ) in vitro in our previous work [13]. However, it was observed in this study that MC induced a significant proliferation in MCF-7 cells in an E-screen assay at the concentration of 0.1 μM, while it did not alter MCF-7 cells proliferation when co-administered with estradiol at all tested concentrations. In addition, MC did not alter ERα and ERβ stably transfected in HEK293T cells when co-administered with estradiol. These observations are consistent with those obtained in the E-screen assay and suggest that MC has a weak estrogen-like effect but no antiestrogenic effects in vitro. The E-screen assay measures cell proliferation, which is well known as a hallmark of estrogen-like activity [23]. The fact that MC induced MCF-7 cell proliferation and failed to transactivate ERs in vitro raises two hypotheses. On one hand, MC might induce its estrogenicity via a non-genomic pathway, since MCF-7 cell proliferation involves both genomic and non-genomic pathways [18]. On the other hand, this compound might undergo hydroxylation by MCF-7 cell enzymes and then bind to ERs such as 6-hydroxycoumarin, which is well known to transactivate ERs [24]. In vivo results show that a 3-day treatment with MC neither induced estrogenic nor antiestrogenic effects on the uterus. However, a slight but not significant increase in endometrial thickness was observed in vivo*,* which is consistent with the weak estrogenicity observed with MC in vitro. The uterus and mammary glands express much ERα than ERβ [25], which may explain the above observations. These results suggest that MC is not responsible for the observed estrogenic effects of *F. umbellata* extract on the uterus [13]. Furthermore, MC induced a significant increase in vaginal epithelial height in ovariectomized rats at the dose of 1 mg/kg after a 3-day treatment. In addition, no estrogenic activity was noted in the uterus after 21 consecutive days of treatment with this compound in the postmenopausal-like rat model, while it induced a significant increase in vaginal epithelial height as it did in the 3-day uterotrophic assay. These results point out a tissue-selective activity for this compound and strengthen our hypothesis that MC is responsible only in part for the estrogenic activity of *F. umbellata.* 6-hydroxycoumarin, Genistein and Biochanin A detected in *F. umbellata* extract by UPLC-MS in this study are well known phytoestrogens, that might account for its effects on the uterus.

After 21 days of treatment with MC and *F. umbellata* aqueous extract, no significant changes were noticed on the relative weights of liver, lungs, kidneys, and adrenal glands as well as in the microarchitecture of the liver, lungs and kidneys; suggesting that these substances are weakly toxic. These observations corroborate our previous finding that the LD50 of *F. umbellata* is more than 5000 mg/kg [13]; thus supporting its use in the traditional medicine. Interestingly, a significant increase in femur wet and dry weights as well as in the tibia wet weight with *F. umbellata* aqueous extract was observed at the dose of 200 mg/kg, while MC failed to induce such effects. Since postmenopausal osteoporosis is a worldwide health problem with a high prevalence [26], these results encourage the exploration of the antiosteoporotic potential of *F. umbellata* extract. Preventive effects of phytoestrogens on postmenopausal osteoporosis are well-known [27]. As far as *F. umbellata* transactivated ERs in vitro, its components might bind to ERs, which have been localized in osteoblasts and osteoclasts and limit bone reabsorption by inhibiting osteoclasts [28]. It seems that MC does not contribute to the effects of *F. umbellata* aqueous extract in bones.

Estrogen deficiency is associated with dyslipidemia, a major cause of increased risk of developing cardiovascular disease [29]. In this study ovariectomy induced a significant variation of serum total cholesterol levels. Following treatment, E2V decreased the serum total cholesterol levels compared to the negative control (OVX). E2 has been shown to inhibit the hydroxymethylglutaryl coenzyme A (HMG-CoA) reductase in cholesterol biosynthesis as well as the rate-limiting enzyme in the cholesterol synthesis de novo [30], hence reducing the synthesis of cholesterol. Scientific reports point out the evidence that this effect on cholesterol metabolism might be mediated by ERα [31]. Conversely, MC failed to change fasting serum lipids. However, *F. umbellata* aqueous extract exhibited estrogen-like effects by decreasing fasting serum total cholesterol and increase serum HDL cholesterol levels at a dose of 200 mg/kg. Atherogenic index was used as an indicator of cardiovascular risks. The increase in HDL-Cholesterol levels in E2V-treated animals observed in this work is in accordance with other reports, which demonstrated an increase in HDL-Cholesterol levels following estrogen treatment in humans [32, 33]. Results on atherogenic risks showed that *F. umbellata* but not MC has beneficial effects on lipid metabolism. This might be due to its ability to activate ERs and induce the transcription of genes involved in lipid metabolism. *F. umbellata* extract was found to contain phytoestrogens (Genistein, Biochanin A, hydroxylcoumarin) which may result from the activation of gene transcription through selective binding of phytoestrogens to ERα and ERβ [34]. Such gene, in turn might display beneficial effects on lipid metabolism in postmenopausal women.

## Conclusion

In conclusion, MC exhibited a weak estrogen activity in vitro as well as in vivo; suggesting that the estrogenic effects displayed by the whole extract is mostly due to the other phytoestrogens present in the plant. However, MC could be beneficial with regard to vaginal dryness as it showed a tissue specific effect without exposing the uterus to a potential tumorigenic growth.

## Additional files

Additional file 1: Table S1. Summary of compounds separated and identified in *F. umbellata* aqueous extract by UHPLC-ESI-HRMS analysis in the negative ion mode. **Figure S1.** Microphotographs of HE-stained sections (400×) of liver, lungs, kidneys and femur from different experimental rat groups in postmenopausal-like condition after 3 weeks of treatment. SHAM = Sham operated rats treated with vehicle as normal control; OVX = Ovariectomized rats treated with vehicle as negative control; E_2_V = Ovariectomized rats treated with estradiol valerate at the dose of dose 1 mg/kg BW as positive control; FU 50 and 200 = Ovariectomized rats treated with *F. umbellata* aqueous extract at the doses of 50 and 200 mg/kg BW, respectively; MC = Ovariectomized rats treated with 7-methoxycoumarin at the dose of 1 mg/kg BW. Vp = portal veine, H = Hepatocyte; S = sinusoids; A = alveol; Ba = Aveolar bag; TB = Trabecular bone; MB = marrow bone; Mi = microglie; Ne = Neurone; Co = Cortex. G = Glomerula; Dt = Distal tube; Pt = Proximal tube. (DOCX 797 kb)

## Abbreviations

C-hex: Cyclo hexane
CNH: Cameroon National Herbarium
CPC: Centrifugal partition chromatography
DCM: Dichloromethane
DMEM: Dulbecco's Modified Eagle Medium
DMSO: Dimethylsulfoxide
ER: Estrogen receptor
F: Fraction
FBS: Fetal bovine serum
FCS: Fetal calf serum
HEPES: 4-(2-hydroxyethyl)-1-piperazineethane sulfonic acid
HPLC: High Performance Liquid Chromatography
MC: 7-methoxycoumarin
MeOH: Methanol
NMR: Nuclear magnetic resonance
PBS: Phosphate-buffered saline
PE: Proliferative effect
RPMI: Roswell Park Memorial Institute
SRB: Sulforhodamine-B
