# Supplementary material for: Estrogen-like and tissue-selective effects of 7-methoxycoumarin from Ficus umbellata (Moraceae): an in vitro and in vivo study
Source: BMC Complement Altern Med. 2017 Aug 2;17:383. doi: 10.1186/s12906-017-1895-9 (PMC5541738; doi:10.1186/s12906-017-1895-9)
Supplement: Supplementary file 1 — Summary of compounds separated and identified in F. umbellata aqueous extract by UHPLC-ESI-HRMS analysis in the negative ion mode. Figure S1. Microphotographs of HE-stained sections (400×) of liver, lungs, kidneys and femur from different experimental rat groups in postmenopausal-like condition after 3 weeks of treatment. SHAM = Sham operated rats treated with vehicle as normal control; OVX = Ovariectomized rats treated with vehicle as negative control; E2V = Ovariectomized rats treated with estradiol valerate at the dose of dose 1 mg/kg BW as positive control; FU 50 and 200 = Ovariectomized rats treated with F. umbellata aqueous extract at the doses of 50 and 200 mg/kg BW, respectively; MC = Ovariectomized rats treated with 7-methoxycoumarin at the dose of 1 mg/kg BW. Vp = portal veine, H = Hepatocyte; S = sinusoids; A = alveol; Ba = Aveolar bag; TB = Trabecular bone; MB = marrow bone; Mi = microglie; Ne = Neurone; Co = Cortex. G = Glomerula; Dt = Distal tube; Pt = Proximal tube. (DOCX 797 kb) [file 12906_2017_1895_MOESM1_ESM.docx]

**Supplementary data 1**. Summary of compounds separated and identified in *F. umbellata* aqueous extract by UHPLC-ESI-HRMS analysis in the negative ion mode.

| **Tr** | **m/z** | **EC** | **ppm** | **DBE** | **Fragments (m/z, EC, DBE)** | **Putative identification** |
| --- | --- | --- | --- | --- | --- | --- |
| **1.07** | 169.0136 | C_7_H_5_O_5_ | -0.6 | 5.5 |  | Trihydroxybenzoic acid |
| **1.76** | 181.0507 | C_9_H_9_O_4_ | 3.3 | 5.5 |  | Dimethoxybenzoic acid |
| **1.89** | 153.0188 | C_7_H_5_O_4_ | 0 | 5.5 |  | gentisic acid |
| **2.09** | 353.0871 | C_16_H_17_O_9_ | -1.6 | 8.5 |  | Caffeoylquinic acid |
| **2.53** | 447.1500  493.1559  [M-H+HCOOH]^-^ | C_18_H_23_O_13_  C_19_H_25_O_15_ | 0.4  -0.7 | 6.5  6.5 | 153.0551, C_7_H_5_O_4_, 5.5  125.0237, C_6_H_5_O_3_, 4.5 | Dihydroxybenzoic acid hexoside pentoside |
| **2.60** | 417.1027  825.2147  [2M-H]^-^ | C_17_H_21_O_12_  C_33_H_45_O_24_ | -1.4  -18.7 | 7.5  11.5 | 285.0595, C_12_H_13_O_8_, 6.5  191.0547, C_7_H_11_O_6_, 2.5  152.0111, C_7_H_7_O_4_, 6  108.0214, C_6_H_4_O_2_, 5  109.0286, C_6_H3O_2_, 4.5 | Dihydroxybenzoic acid di-pentoside |
| **2.83** | 181.0496  167.0702 | C_9_H_9_O_4_  C_9_H_11_O_3_ | -2.5  -3.6 | 5.5  4.5 | 137.0602, C8H9O2, 4.5  121.0279, C7H5O2, 5.5 | Phenolic acid derivative |
| **3.65** | 357.1185  715.2444 [2M-H]^-^ | C_16_H_21_O_9_  C_32_H_43_O_8_ | -0.3  -0.7 | 6.5  11.5 | 195.0657, C_10_H_11_O_4_, 5.5  151.0757, C_9_H_11_O_2_, 4.5 | dihydroferulic acid hexoside |
| **3.95** | 355.1031 | C_16_H_19_O_9_ | 0.6 | 5.5 | 193.0500, C_10_H_9_O_4_, 6.5  149.0606, C_9_H_9_O_2_, 5.5  133.0293, C_8_H_5_O_2_, 6.5 | Ferulic acid hexoside |
| **6.0** | 175.0384 | C_10_H_7_O_3_ | -6.3 | 7.5 |  | 7-methoxycoumarin |
| **6.35** | 177.0184 | C_9_H_5_O_4_ | -2.3 | 7.5 | 149.0615, C_9_H_9_O_2_, 5.5  105.0331, C_7_H_5_O, 5.5 | dihydroxycoumarin |
| **6.55** | 269.0446 | C_15_H_9_O_5_ | -1.5 | 11.5 | 229.0853, C_14_H_13_O_3_, 8.5  201.0569, C_12_H_19_O_3_, 8.5  195.0634, C_10_H_11_O_4_, 5.5  174.0309, C_10_H_6_O_3_, 8  159.0461, C_10_H_7_O_2_, 7.5  141.0319, C_10_H_5_O, 8.5  133.0275, C_8_H_5_O_2_, 6.5  107.0136, C_6_H_3_O_2_, 5.5 | Genistein |
| **7.34** | 329.2324 | C_18_H_33_O_5_ | -1.2 | 2.5 | 195,0653, C10H11O4, 5.5  171.1022, C_9_H_15_O_3_, 2.5  151,0754, C_9_H_11_O_2_, 4.5 | trihydroxy-octadecenoic acid (329) |
| **8.89** | 283.0607 | C_16_H_11_O_5_ | 0.4 | 11.5 | 268.0368, C_15_H_8_O_5_, 12  239.0341, C_14_H_7_O_4_, 11.5  177.0565, C_10_H_9_O_3_, 6.5  149.0563, C_9_H_9_O_2_, 5.5  132.0221, C_8_H_4_O_2_, 7 | Génistein 4’ methyl ether (biochanin A) |
| **9.01** | 229.0865 | C_14_H_13_O_3_ | 0 | 8.5 | 213.0555, C_13_H_9_O_3_, 9.5  174.0315, C_10_H_6_O_3_, 8  146.0366, C_9_H_6_O2, 7  130.0418, C_9_H_6_O, 7 | Prenyl-7-hydroxycoumarin |
| **12.90** | 293.2116 | C_18_H_29_O_3_ | -0.3 | 4.5 |  | Hydroxy octadecatrienoic acid |
| **13.92** | 295.2274  591.4633 [2M-H]^-^ | C_18_H_31_O_3_  C_36_H_63_O_6_ | 0.3  1.4 | 3.5  5.5 |  | Hydroxy octadecadienoic acid |
| **14.33** | 455.3524  911.7121 [2M-H]^-^ | C_30_H_47_O_3_ | -0.2 | 7.5 |  | Triterpen derivative |
| **14.71** | 271.2273  543.4625 [2M-H]^-^ | C_16_H_31_O_3_ | 0 | 1.5 |  | hydroxy hexadecanoic acid |

|  | **SHAM** | **OVX** | **E2V** | **FU 50** | **FU 200** | **MC** |
| --- | --- | --- | --- | --- | --- | --- |
| **Liver** | 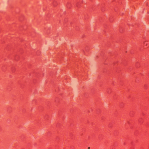 **S**  **H**  **Vp** | 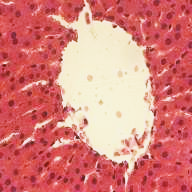 **S**  **H**  **Vp** | 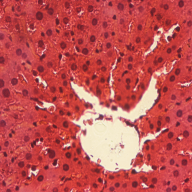 **Vp**  **H**  **S** | 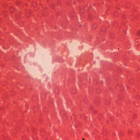 **Vp**  **H**  **S**  **S** | 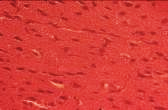 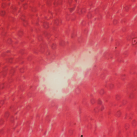 **Vp**  **H**  **S**  **S** | 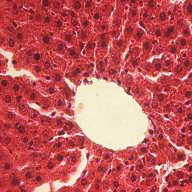 **Vp**  **H**  **S**  **S** |
| **Lung** | 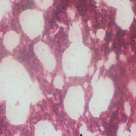 **Sa**  **ALv** | 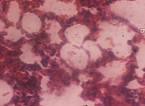 **Sa**  **A** | 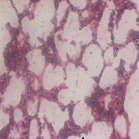 **Sa**  **A** | 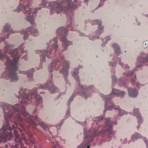 **Sa**  **A** | 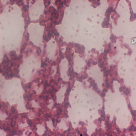 **Sa**  **A** | 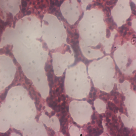 **Sa**  **A** |
| **Kidney** | **G** 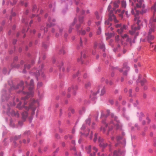 **G**  **Dt**  **G** | **Dt** 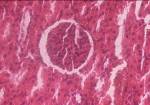 **G** | 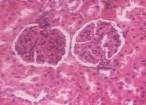 **G**  **Dt**  **G** | **Dt** 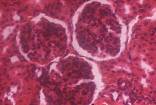 **G**  **G**  **G** | 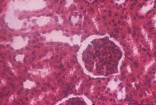 **Dt**  **G** | **G** 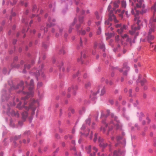 **G**  **Dt**  **G** |
| **Femur** | 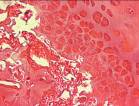 **TB**  **MB** | 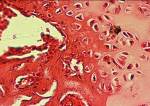 **TB**  **MB** | 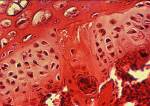 **MB**  **TB** | 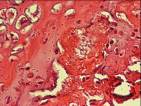 **MB**  **TB** | 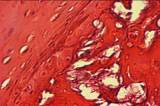 **MB**  **TB** | 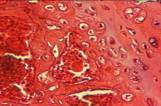 **MB**  **TB** |

**Supplementary data 2**.
